# Effects of Three Calcium Silicate Cements on Inflammatory Response and Mineralization-Inducing Potentials in a Dog Pulpotomy Model

**DOI:** 10.3390/ma11060899

**Published:** 2018-05-27

**Authors:** Chung-Min Kang, Jiwon Hwang, Je Seon Song, Jae-Ho Lee, Hyung-Jun Choi, Yooseok Shin

**Affiliations:** 1 Department of Pediatric Dentistry, College of Dentistry, Yonsei University, Seoul, 03722, Korea; zezu7@yuhs.ac (C.-M.K.); kntdent@naver.com (J.H.); songjs@yuhs.ac (J.S.S.); leejh@yuhs.ac (J.-H.L.); choihj88@yuhs.ac (H.-J.C.); 2 Department of Pharmacology, College of Medicine, Yonsei University, Seoul 03722, Korea; 3 Oral Science Research Center, College of Dentistry, Yonsei University, Seoul 03722, Korea; 4 Department of Conservative Dentistry, College of Dentistry, Yonsei University, 50-1 Yonseiro, Seodaemun-Gu, Seoul 03722, Korea

**Keywords:** calcium silicate cements, pulpal response, mineralization, calcific barrier, inflammation, odontoblastic layer

## Abstract

This beagle pulpotomy study compared the inflammatory response and mineralization-inducing potential of three calcium silicate cements: ProRoot mineral trioxide aggregate (MTA) (Dentsply, Tulsa, OK, USA), OrthoMTA (BioMTA, Seoul, Korea), and Endocem MTA (Maruchi, Wonju, Korea). Exposed pulp tissues were capped with ProRoot MTA, OrthoMTA, or Endocem MTA. After 8 weeks, we extracted the teeth, then performed hematoxylin-eosin and immunohistochemical staining with osteocalcin and dentin sialoprotein. Histological evaluation comprised a scoring system with eight broad categories and analysis of calcific barrier areas. We evaluated 44 teeth capped with ProRoot MTA (*n* = 15), OrthoMTA (*n* = 18), or Endocem MTA (*n* = 11). Most ProRoot MTA specimens formed continuous calcific barriers; these pulps contained inflammation-free palisading patterns in the odontoblastic layer. Areas of the newly formed calcific barrier were greater with ProRoot MTA than with Endocem MTA (*p* = 0.006). Although dentin sialoprotein was highly expressed in all three groups, the osteocalcin expression was reduced in the OrthoMTA and Endocem MTA groups. ProRoot MTA was superior to OrthoMTA and Endocem MTA in all histological analyses. ProRoot MTA and OrthoMTA resulted in reduced pulpal inflammation and more complete calcific barrier formation, whereas Endocem MTA caused a lower level of calcific barrier continuity with tunnel defects.

## 1. Introduction

Vital pulp therapy consists of apexogenesis, pulpotomy, pulpal debridement, indirect pulp capping, and direct pulp capping [1]. The aim of these treatments includes maintenance of vitality and preservation of the remaining pulp to enable adequate healing of the pulp-dentin complex [2,3]. These procedures offer a more conservative approach to root canal therapy for traumatic and cariously exposed pulps [4,5]. ProRoot mineral trioxide aggregate (PMTA) (Dentsply, Tulsa, OK, USA) has been recognized as an appropriate material for vital pulp therapy, and has provided higher clinical success rates in a long-term study [6].

Although PMTA possesses bioactive and antibacterial activities with high sealing properties [7,8,9,10], it has some drawbacks such as a prolonged setting time, discoloration potential, and difficult handling properties [11]. Recently, calcium silicate cement development has helped to overcome these disadvantages in the search for bioactive dental materials. These include OrthoMTA (OMTA; BioMTA) and Endocem MTA (EMTA; Maruchi, Wonju, Korea).

OMTA contains lower concentrations of heavy metals for greater biocompatibility [12] and has a low expansion rate and a good sealing ability [13]. According to the manufacturer, OMTA prevents microleakage by forming a layer of hydroxyapatite that acts as an interface between the material and the canal wall [14]. Moreover, it has been reported to create a favorable environment for bacterial entombment through intratubular mineralization [15]. Clinical studies reported that both PMTA and OMTA had high success rates in pulpotomy and partial pulpotomy when used in primary and permanent teeth [16,17]. However, OMTA showed lower biocompatibility compared with PMTA in an in vitro study [18]. EMTA has been introduced as an MTA-derived pozzolan cement. EMTA has a short setting time (around 4 min) without the addition of a chemical accelerator because it uses a pozzolanic reaction [19]. It is convenient to handle based on its adequate consistency and washout resistance [20]. Indeed, EMTA demonstrated good biocompatibility, osteogenicity, and odontogenic effects similar to PMTA in an in vitro study [19].

Despite the increased use of various calcium silicate cements in vital pulp therapy, their effects after pulpotomy in an in vivo animal model have not yet been investigated. In addition, most studies have had short durations of approximately 4 weeks. Accordingly, the present study aimed to evaluate and compare levels of calcific barrier formation, inflammation reaction, and hard tissue barrier formation histologically following the application of PMTA, OMTA, and EMTA for 8 weeks in a beagle pulpotomy model. The null hypothesis is that there is no significant difference in inflammatory response and mineralization-inducing potentials among the three materials; both OMTA and EMTA have similar biologic effects to PMTA.

## 2. Materials and Methods

### 2.1. Animal Model

The present study used a beagle dog pulpotomy model. The inclusion criteria were non-damaged dentition and healthy periodontium. The teeth of two dogs were numbered and randomly divided into three groups with a table of random sampling numbers; the three calcium silicate material groups are shown in Table 1. We performed animal selection, control, the surgical operation, and preparation as per the procedures approved by the Yonsei University Health System’s Institutional Animal Care and Use Committee (certification #2013-0317-4). These experiments were performed in accordance with the National Institutes of Health Guide for the Care and Use of Laboratory Animals.

### 2.2. Surgical Procedure

We performed all operations in a clean, sterilized room. We administered intravascular injections of tiletamine/zolazepam (Zoletile^®^ 5 mg/kg; Virbac Korea, Seoul, Korea) and xylazine (Rompun^®^ 0.2 mg/kg; Bayer Korea, Seoul, Korea), and used the inhalational anesthetic isoflurane (Gerolan^®^, Choongwae Pharmaceutical, Seoul, Korea) for general anesthesia induction. To prevent infection, we injected enfloxacin (5 mg/kg) subcutaneously immediately before and after the operation. We administered amoxicillin clavulanate (12.5 mg/kg) orally for 5 to 7 days after the operation.

### 2.3. Pulpotomy Procedure

We used lidocaine hydrochloride (2%) with 1:100,000 epinephrine (Kwangmyung Pharmaceutical, Seoul, Korea) for local anesthesia. After forming a cavity on the occlusal surface using a high-speed carbide bur #330 (H7 314 008, Brasseler, Lemgo, Germany), we mechanically exposed the pulp. We removed the crown of the pulp at the level of the cementoenamel junction and stopped bleeding by injecting sterile saline and applying slight pressure with sterile cotton pellets. We applied the MTA to the top of the cut pulp as per the manufacturer’s guidelines in each group. When we applied the MTA to the pulp area, we used cotton balls soaked in saline. We performed the final cavity restoration using the self-curing glass ionomer cement Ketac-Molar (3M ESPE; St. Paul, MN, USA). Eight weeks after the operation, we euthanized the dogs by over-sedation.

### 2.4. Histological Analysis

We extracted the teeth with forceps and removed one-third of the root using a high-speed bur. We fixed the specimens in 10% neutral-buffered formalin (Sigma-Aldrich, St. Louis, MO, USA) for 48 h, and demineralized them in ethylenediaminetetraacetic acid (EDTA; pH 7.4; Fisher Scientific, Houston, TX, USA) for 6 weeks before embedding them in paraffin. For each specimen, we created 3-µm continuous sections in the buccolingual direction and stained them subsequently with hematoxylin and eosin (HE). We used InnerView 2.0 software (InnerView Co., Seongnam-si, Gyeonggi-do, Korea) for image analysis. Five observers blinded to the group allocations examined the specimens. We evaluated the specimens for calcific barrier formation, dental pulp inflammation, and odontoblastic cell layer formation with a scoring system reported in a previous study [21,22,23] (Table 2). We adopted the score agreed upon by at least three of the five observers who were blinded to specimen grouping. In addition, we measured the area of the newly formed hard tissue using ImageJ version 1.48 (National Institute of Health, Bethesda, MD, USA).

### 2.5. Immunohistochemistry

For immunohistochemistry (IHC), we deparaffinized 3-μm cross-sections with xylene, rehydrated them, and rinsed them with distilled water. For antigen retrieval, we used protease K (Dako North America Inc., Carpinteria, CA, USA) for osteocalcin (OC) and dentin sialoprotein (DSP) staining. To activate endogenous peroxidase, we added 3% hydrogen peroxide, while preventing non-specific binding by incubating sections in 5% bovine serum albumin (Sigma-Aldrich). Subsequently, we incubated sections overnight with the following primary antibodies: anti-OC antibody (rabbit polyclonal, Ab109112, 1:10,000; Abcam, Cambridge, UK) or anti-DSP antibody (rabbit polyclonal, sc-33586, 1:500; Santa Cruz Biotechnology, Santa Cruz, CA, USA). Subsequently, we applied EnVision + System-Horseradish peroxidase (HRP)-Labeled Polymer anti-rabbit (K4003, Dako) for 20 min. After developing color using the labeled streptavidin biotin kit (Dako) as per the manufacturer’s guidelines, we counterstained the sections with Gill’s hematoxylin (Sigma-Aldrich).

### 2.6. Statistical Analysis

We performed statistical analyses using SPSS version 23 software (SPSS, Chicago, IL, USA). To analyze the area of the newly formed calcific barrier, we applied a one-way analysis of variance (ANOVA) (significance at *p* < 0.05) and the post hoc Scheffé test (Bonferroni correction; *p* < 0.017).

## 3. Results

The present study used two male beagle dogs aged 18–24 months that weighed approximately 12 kg. We selected 60 teeth from the two dogs. Among the 60 teeth, we excluded five teeth from the PMTA group, two from the OMTA group, and nine from the EMTA group that failed during tooth removal or specimen production; we evaluated only 44 specimens in the final analysis. Eventually, we analyzed the PMTA (*n* = 15), OMTA (*n* = 18), and EMTA (*n* = 11) specimens histologically.

### 3.1. Calcific Barrier Formation

**Figure 1:**
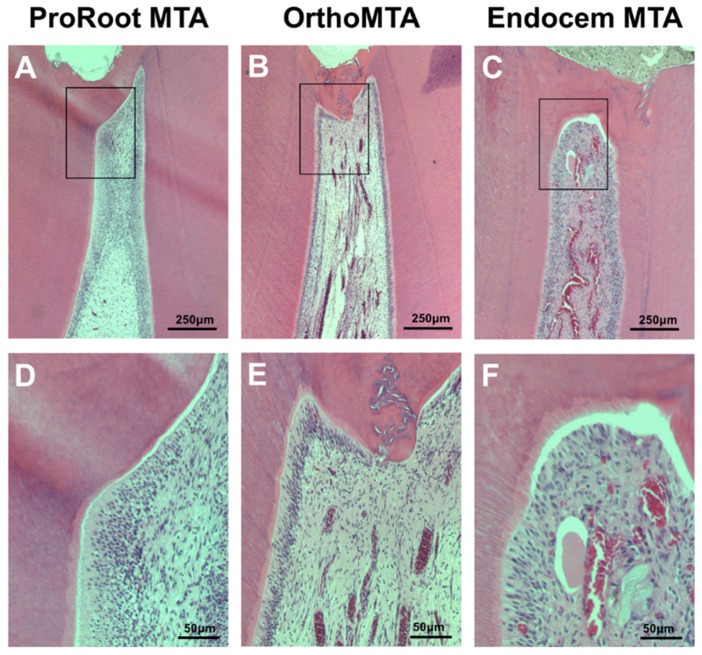
Hematoxylin-eosin staining for the evaluation of the histomorphologic characteristics of the newly formed calcific barrier (CB) after 8 weeks. Most PMTA and OMTA specimens formed continuous CBs and the pulps contained palisading patterns in the odontoblastic layer that were free from inflammation. However, EMTA specimens showed less favorable odontoblastic layer formation ((**A**–**C**): scale bars = 250 µm, (**D**–**F**): scale bars = 50 µm).

**Figure 2:**
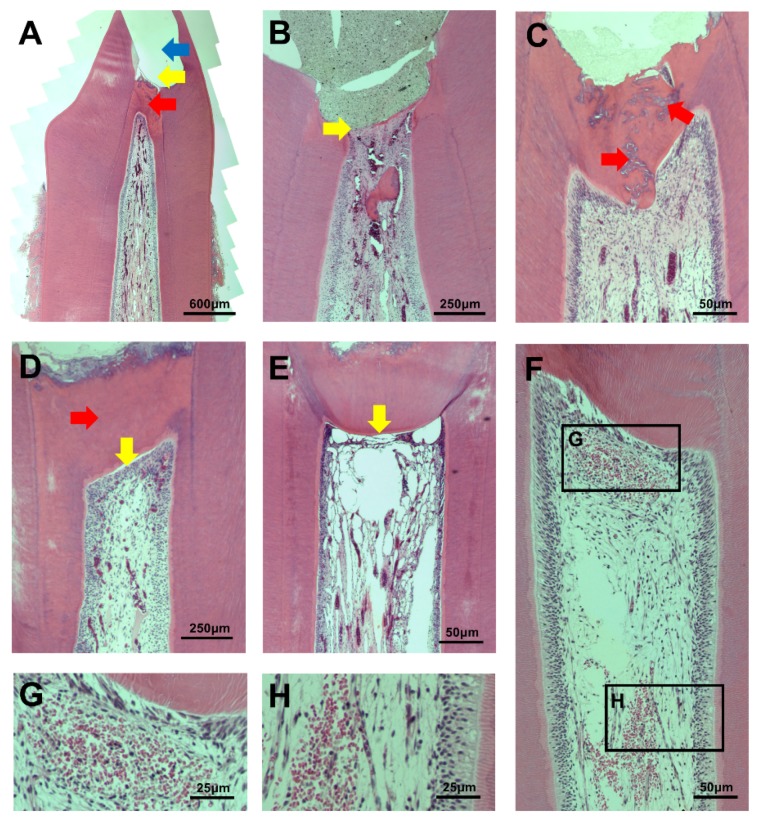
(**A**) An example of a pulpotomy procedure. The site of pulpotomy (yellow arrow) was an upper part of the calcific barrier (red arrow). MTA was filled in the pulp exposure area, but the material is not visible due to partial loss during specimen processing (blue arrow); (**B**) An EMTA specimen without a calcific barrier (yellow arrow). This corresponds to score 4 in the calcific barrier continuity category; (**C**) Moderate to severe tubule formation in calcific barrier of an OMTA specimen (red arrow); (**D**) A PMTA specimen with complete calcific barrier formation and no tubule in barrier (red arrow). It also has complete palisading cell pattern in odontoblastic cell layer (yellow arrow); (**E**) An EMTA specimen with absent odontoblastic cell layer under calcific barrier. This corresponds to score 4 in the odontoblastic cell layer category; (**F**–**H**) Inflammatory cells that are observed in coronal and middle pulps.

**Figure 3:**
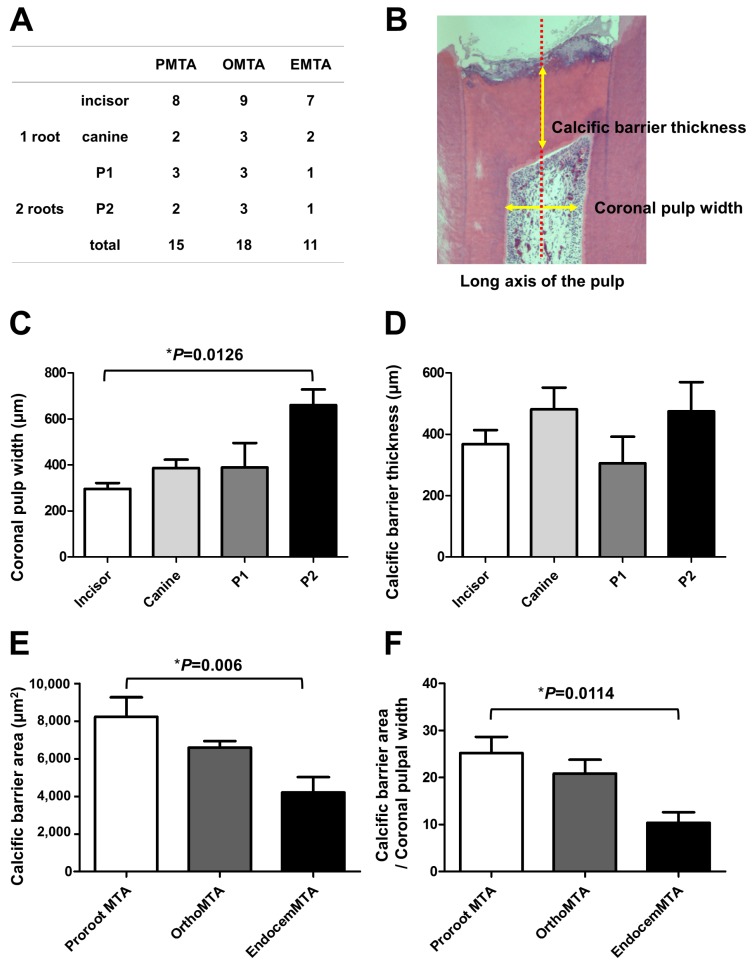
The area of the newly formed calcific barrier for each material after 8 weeks. (**A**) The distributions of tooth types among the three test groups; (**B**) An example measuring coronal pulpal width and calcific barrier thickness; Although the thickness of the calcific barriers did not vary according to tooth type (**C**); the width of the coronal pulp differed by tooth type (*p* = 0.0126) (**D**). Thus, the area of the calcific barrier was calculated by dividing by the horizontal width of the coronal pulp. The bars represent the mean ± the standard deviation; (**E**) In the PMTA group, the calcific barrier is widest, followed by those in the OMTA and EMTA groups. There is a statistically significant difference between the PMTA and EMTA groups (*p* = 0.006); (**F**) When the calcific barriers were standardized by coronal pulpal width, PMTA also had a significantly higher area than EMTA (*p* = 0.0114). We performed statistical analyses with a one-way ANOVA and the post hoc Scheffé test. The number of specimens is *n* = 15 in the PMTA group, *n* = 18 in the OMTA group, and *n* = 11 in the EMTA group.

Most specimens in the PMTA group formed a complete calcific barrier, while some in the OMTA and EMTA groups produced a partially discontinuous calcific barrier (Figure 1 and Table 3). We observed no calcific barrier in only one specimen from the EMTA group (Figure 2B). In addition, the PMTA group produced hard tissue most similar to the dentin, while we observed a partially irregular or thinly formed calcific layer in the OMTA and EMTA groups. Although three groups contained calcific barriers with low tubule formation (Figure 2D), moderate to severe tubule formation in calcific barrier was found in an OMTA specimen (Figure 2C). We compared the areas of the formed calcific barriers; the calcific barrier in the PMTA group was the widest, followed by those in the OMTA and EMTA groups. There was a statistically significant difference between the PMTA and EMTA groups (*p* = 0.006; Figure 3E). When the calcific barriers were standardized with coronal pulpal width according to tooth type, PMTA also had a significantly higher area than EMTA (*p* = 0.0114; Figure 3F).

### 3.2. Pulpal Reaction

We observed mononuclear inflammatory cells in 26.67% of the specimens from the PMTA group, 44.44% from the OMTA group, and 63.64% from the EMTA group (Table 4). When we compared the extent of inflammation among the groups, inflammation was mild or almost absent in the PMTA and OMTA groups. In 9% of the EMTA specimens, we observed inflammatory cells in one-third or more of the coronal pulp or middle pulp. While there was almost no acute inflammation in any group, a few cases demonstrated chronic inflammation exclusively. The pulpal congestion reaction was mild in the PMTA group and most severe in the EMTA group. We observed no pulpal congestion above the moderate level in any of the three groups.

### 3.3. Odontoblastic Cell Layer

In the PMTA group, a complete palisading cell pattern was visible in 60% of the specimens, with 26.67% showing Partial/incomplete palisading cell pattern (Table 5). The OMTA and EMTA groups showed less favorable results compared with the PMTA group: all OMTA specimens showed an odontoblastic cell layer. In contrast, approximately 9.09% of the EMTA group specimens showed no odontoblastic cell layer.

### 3.4. Immunohistochemistry

**Figure 4:**
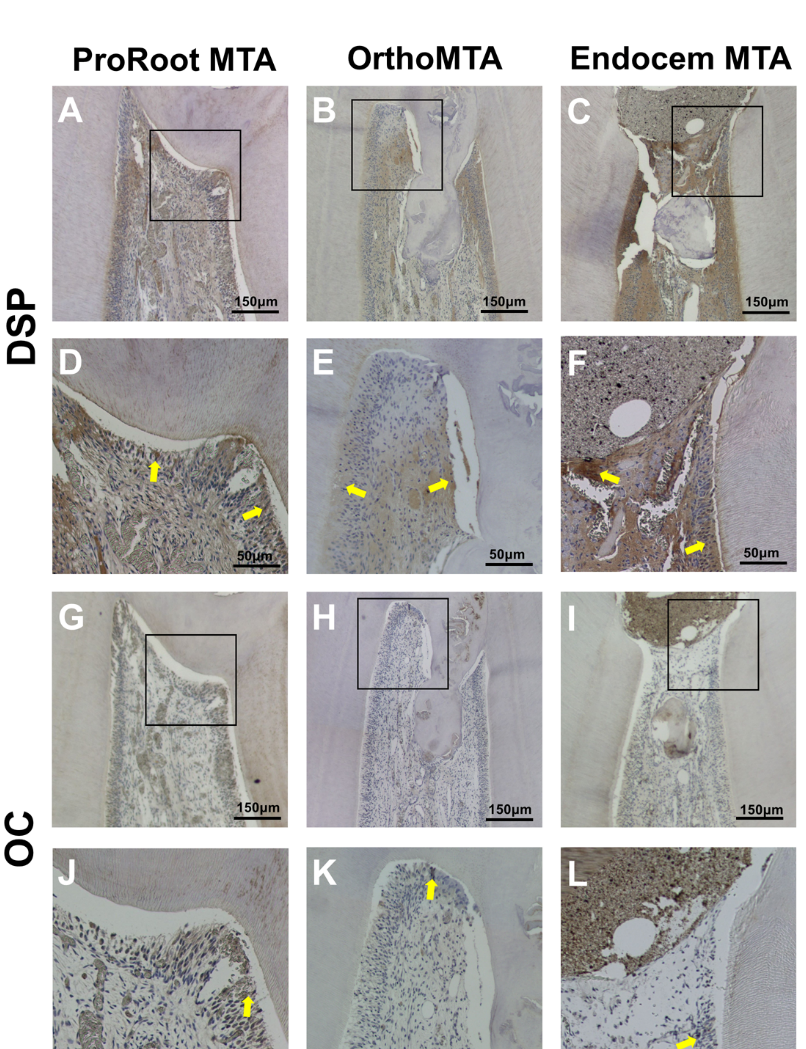
Immunohistochemical staining of dentin sialoprotein (DSP) and osteocalcin (OC). The DSP is highly expressed in all three groups. Although OC is expressed most clearly in odontoblast-like cells in the PMTA group, its expression was reduced in the EMTA group. Yellow arrows indicate cells with a positive signal. ((**A**–**C**,**G**–**I**): scale bars = 150 µm; (**D**–**F**,**J**–**L**): scale bars = 50 µm).

DSP and OC staining indicated hard tissue formation in all three groups. The DSP was highly expressed in all three groups, which indicated odontogenic differential potential (Figure 4A–F). Although OC was also expressed in all three groups, its expression was relatively less in the EMTA group (Figure 4G–L).

## 4. Discussion

Many attempts have been made to improve the clinical application of MTA by modifying chemical components or adding a setting accelerator; however, it is important to note that changes in MTA components may cause adverse effects with respect to physical and biological properties [24]. We recommend that in vivo studies explore the pulpal response when using MTA for vital pulp therapy. We recently reported the pulpal responses to pulpotomy with RetroMTA, TheraCal (Bisco Inc., Schamburg, IL, USA), and PMTA [22], and compared the biological efficacies of Endocem Zr (Maruchi, Wonju, Korea) and PMTA at 4 weeks in an in vivo study [23]. We performed this study to compare and evaluate the pulpal response associated with PMTA, OMTA, and EMTA over 8 weeks in a beagle dog pulpotomy model. To our knowledge, this is the first study to examine the biological response from OMTA compared with those from EMTA and PMTA simultaneously in an in vivo model.

Overall, both PMTA and OMTA showed favorable pulpal reactions and formed an almost complete calcific barrier. EMTA induced a slightly greater inflammatory reaction than the other two MTAs and had a lower amount of calcific barrier formation with tunnel defects. These results suggested that EMTA had relatively less biocompatibility than PMTA or OMTA. In addition, PMTA and OMTA formed favorable palisading patterns in odontoblast cells, which demonstrate that these materials had stronger odontogenic differentiation potential.

We suggest several reasons for the low biocompatibility of EMTA observed in this study. A study reported that EMTA resulted in lower cell viability than PMTA immediately after mixing because of its high pH and the heat from the cement surface [25]. This initial cytotoxic effect might contribute to denaturation of adjacent cells [26]. EMTA was also known to express a lower level of osteopontin, a specific bone mineralization marker, compared with other MTAs [27]. Reduced osteopontin production could be a result of lower hard tissue formation. However, these results were not in accordance with the results of a previous in vitro study, which reported that OMTA was significantly more cytotoxic than PMTA and EMTA [27]. The difference may result from interactions with living cells; chemical reactions in the dentin-MTA interfacial layer might have caused the differences in results between the in vivo and in vitro study models. In addition, while PMTA and OMTA exhibit similar components, EMTA contains different chemicals, such as small particles of pozzolan. A pozzolan is a siliceous and aluminous material that reacts chemically with calcium hydroxide to form calcium silicate hydrate in the presence of water [28]. Unlike the initially high pH levels after mixing, EMTA showed a significantly lower pH value than PMTA and OMTA [29]. A previous study found that an acidic environment adversely affected the physical properties and hydration behavior of MTA [30]. We recommend further study because EMTA had inferior cell viability and a low pH level after setting, but was not significantly different from the other two MTAs.

Despite the small number of reports regarding OMTA and EMTA, various in vivo and clinical studies have demonstrated successful formation of calcific barriers in vital pulp therapy with PMTA [16,17,22,23,31,32]. It is still controversial whether calcific barrier formation at the interface between the pulp and material indicates the success of the treatment. Therefore, to judge the efficacy of different MTAs, it is important to analyze the presence or absence of inflammation (the type and severity) and the continuity, structure, and tubule formation of the formed calcific barrier [33]. This study interpreted the calcific barrier as a sign of healing and a positive reaction to stimulation. In the three MTA groups, the calcific barrier interfaces had columnar cells projecting into the bridge with polarized nuclei, which indicates the formation of odontoblast cells and reparative dentin synthesis. This study also confirmed odontogenic properties with IHC by using OC and DSP. OC is a specific marker of the late odontoblastic development pathway and DSP is a marker of odontoblast regulation in reparative dentin mineralization [34]. In regards to several molecular mechanisms, in vitro studies with PMTA showed continuously increasing transcription of mRNA for DSP [35]. Further, upregulation of OC mRNA confirmed the odontoblastic pathway of cell differentiation, indicating that cells enter a quiescent phase in another calcium silicate cement, Biodentine [36]. IHC revealed that PMTA had the potential to induce greater odontoblastic differentiation than OMTA or EMTA. In the analysis of the mean calcific barrier areas, the areas of newly formed calcific barrier were significantly greater in the PMTA group than in the EMTA group. When comparing the area of newly formed calcific barrier in this study statistically, the size of the pulp would be different according to the type of tooth. Although we tried to distribute the tooth types evenly among the experimental groups, we excluded some specimens during tooth removal and specimen production, especially in the EMTA group. Further, the PMTA was regarded as a positive control, but there was no negative control group in this experiment. We recommend further study with a larger sample and the inclusion of a negative control.

In the present study, we evaluated the pulpal response in pulpotomy model for a period of 8 weeks. Other studies used the same time interval [37,38]. In one study, osteodentin matrix formation occurred during the first 2 weeks; after 3 weeks, a complete layer of reparative dentin was formed at the capping site [39]. Another study reported the presence of a calcified bridge in all specimens at 5 weeks after capping with MTA [34]. Notably, we designed the study to compare pulpal responses in a previous study with a shorter time interval (4 weeks) to determine whether there was any difference in response, compared with an 8-week interval. At 8 weeks, hard tissue formation at the exposure site was thicker with less inflammation than in our previous in vivo study at 4 weeks [22,23].

The results of the animal model may not correspond with those of human teeth. A complete hard tissue barrier appeared 1 week after pulp capping in canine teeth [33], whereas the initiation of hard tissue formation has been reported to start as early as 2 weeks after pulp capping in humans [40]. Most studies reported that it took 30 to 42 days to form a hard tissue barrier in humans [41,42]. In addition, we performed the evaluation of the pulpal response on healthy, intact teeth from dogs. Therefore, these results do not necessarily reflect the effects of newly developed MTAs on inflamed pulps. We advise clinicians to place a wet cotton pellet over the MTA in the first visit, followed by replacement with a permanent restoration at the second visit. In this experiment under G/A, the long setting time of PMTA and OMTA was a limitation. Accordingly, careful consideration is necessary when adapting these results to clinical situations.

In conclusion, the results of the present in vivo study demonstrated that PMTA and OMTA had favorable outcomes when used as pulpotomy materials. Both materials showed biocompatibility and induced high-quality calcific barrier formation at the interface with the pulp tissue. However, EMTA has appropriate characteristics and clinical advantages of a shorter setting time and no tooth discoloration. Regarding the application of these results on pulpotomy in healthy canine pulps with no inflammation, we recommend further clinical studies using human teeth for an evaluation of the biological efficacy of these materials.

## Figures and Tables

**Table 1:** The chemical compositions of the calcium silicate cements tested in this study.

Materials	Composition	Setting Time
PMTA	Tricalcium silicate	Initial setting time: 78 min (±5 min) Final setting time: 261 min (±21 min)
	Tricalcium aluminate	
	Dicalcium silicate	
	Tetracalcium aluminoferrite	
	Gypsum	
	Free calcium oxide	
	Bismuth oxide	
OMTA	Tricalcium silicate	Initial setting time: 180 min Final setting time: 360 min (±21 min)
	Dicalcium silicate	
	Tricalcium aluminate	
	Tetracalcium aluminoferrite	
	Free calcium oxide	
	Bismuth oxide	
EMTA	Calcium oxide	Initial setting time: 2 min (±30 s) Final setting time: 4 min (±30 s)
	Silicon dioxide	
	Bismuth oxide	
	Aluminum oxide	
	H_2_O/CO_2_	
	Magnesium oxide	
	Sulfur trioxide	
	Ferrous oxide	
	Titanium dioxide	

PMTA, ProRoot MTA^®^ (Dentsply Tulsa, OK, USA); OMTA, Ortho MTA^®^ (BioMTA, Seoul, Korea); EMTA, Endocem MTA^®^ (Maruchi, Wonju, Korea).

**Table 2:** Scores used during the histological analysis of calcific barriers and dental pulp.

**Scores**	**Calcific Barrier Continuity**
**1**	Complete calcific barrier formation
**2**	Partial/incomplete calcific barrier formation extending to more than one-half of the exposure site but not completely closing the exposure site
**3**	Initial calcific barrier formation extending to no more than one-half of the exposure site
**4**	No calcific barrier formation
**Scores**	**Calcific barrier morphology**
**1**	Dentin or dentin-associated with irregular hard tissue
**2**	Only irregular hard tissue deposition
**3**	Only a thin layer of hard tissue deposition
**4**	No hard tissue deposition
**Scores**	**Tubules in calcific barrier**
**1**	No tubules present
**2**	Mild (tubules present in less than 30% of the calcific barrier)
**3**	Moderate to severe (tubules present in more than 30% of the calcific barrier)
**4**	No hard tissue deposition
**Scores**	**Inflammation intensity**
**1**	Absent or very few inflammatory cells
**2**	Mild (an average of <10 inflammatory cells)
**3**	Moderate (an average of 10–25 inflammatory cells)
**4**	Severe (an average >25 inflammatory cells)
**Scores**	**Inflammation extensity**
**1**	Absent
**2**	Mild (inflammatory cells next to the dentin bridge or area of pulp exposure only)
**3**	Moderate (inflammatory cells observed in one-third or more of the coronal pulp or in the mid pulp)
**4**	Severe (all of the coronal pulp is infiltrated or necrotic)
**Scores**	**Inflammation type**
**1**	No inflammation
**2**	Chronic inflammation
**3**	Acute and chronic inflammation
**4**	Acute inflammation
**Scores**	**Dental pulp congestion**
**1**	No congestion
**2**	Mild (enlarged blood vessels next to the dentin bridge or area of pulp exposure only)
**3**	Moderate (enlarged blood vessels observed in one-third or more of the coronal pulp or in the mid pulp)
**4**	Severe (all of the coronal pulp is infiltrated with blood cells)
**Scores**	**Odontoblastic cell layer**
**1**	Complete palisading cell pattern
**2**	Partial/incomplete palisading cell pattern
**3**	Presence of odontoblast-like cells only
**4**	Absent

**Table 3:** Score percentages for calcific barriers.

**Groups**	**Calcific Barrier Continuity (%)**			
	**1**	**2**	**3**	**4**
PMTA	100 (15/15) *	-	-	-
OMTA	66.67 (12/18)	16.67 (3/18)	16.67 (3/18)	-
EMTA	45.45 (5/11)	18.18 (2/11)	27.27 (3/11)	9.09 (1/11)
**Groups**	**Calcific Barrier Morphology (%)**			
	**1**	**2**	**3**	**4**
PMTA	86.67 (13/15)	13.33 (2/15)	-	-
OMTA	38.89 (7/18)	27.78 (5/18)	33.33 (6/18)	-
EMTA	45.45 (5/11)	18.18 (2/11)	27.27 (3/11)	9.09 (1/11)
**Groups**	**Tubules in Calcific Barrier (%)**			
	**1**	**2**	**3**	**4**
PMTA	60 (9/15)	33.33 (5/15)	6.67 (1/15)	-
OMTA	61.11 (11/18)	27.78 (5/18)	11.11 (2/18)	-
EMTA	63.64 (7/11)	18.18 (2/11)	9.09 (1/11)	9.09 (1/11)

PMTA, ProRoot MTA^®^; OMTA, Ortho MTA^®^; EMTA, Endocem MTA^®^. * (number of teeth receiving the score/total number of teeth evaluated).

**Table 4:** Score percentages for inflammatory responses.

**Groups**	**Inflammation Intensity (%)**			
	**1**	**2**	**3**	**4**
PMTA	73.33 (11/15) *	26.67 (4/15)	-	-
OMTA	55.56 (10/18)	44.44 (8/18)	-	-
EMTA	36.36 (4/11)	63.64 (7/11)	-	-
**Groups**	**Inflammation Extensity (%)**			
	**1**	**2**	**3**	**4**
PMTA	73.33 (11/15)	26.67 (4/15)	-	-
OMTA	55.56 (10/18)	44.44 (8/18)	-	-
EMTA	36.36 (4/11)	54.55 (6/11)	9.09 (1/11)	-
**Groups**	**Inflammation Type (%)**			
	**1**	**2**	**3**	**4**
PMTA	73.33 (11/15)	26.67 (4/15)	-	-
OMTA	55.56 (10/18)	44.44 (8/18)	-	-
EMTA	36.36 (4/11)	63.64 (7/11)	-	-
**Groups**	**Dental Pulp Congestion (%)**			
	**1**	**2**	**3**	**4**
PMTA	40 (6/15)	53.33 (8/15)	6.67 (1/15)	-
OMTA	27.78 (5/18)	61.11 (11/18)	11.11 (2/18)	-
EMTA	18.18 (2/11)	63.64 (7/11)	18.18 (2/11)	-

* (number of teeth receiving the score/total number of teeth evaluated).

**Table 5:** Score percentages for the odontoblastic cell layer.

Groups	Odontoblastic Cell Layer (%)			
	1	2	3	4
PMTA	60 (9/15) *	26.67 (4/15)	13.33 (4/15)	-
OMTA	33.33 (6/18)	50 (9/18)	16.67 (3/18)	-
EMTA	45.45 (5/11)	18.18 (2/11)	27.27 (3/11)	9.09 (1/11)

* (number of teeth receiving the score/total number of teeth evaluated).
